# A novel epithelial‐mesenchymal transition molecular signature predicts the oncological outcomes in colorectal cancer

**DOI:** 10.1111/jcmm.16387

**Published:** 2021-03-04

**Authors:** Zezhi Shan, Wen Wu, Xuebing Yan, Yongzhi Yang, Dakui Luo, Qi Liu, Xinxiang Li, Ajay Goel, Yanlei Ma

**Affiliations:** ^1^ Department of Colorectal Surgery Fudan University Shanghai Cancer Center Shanghai China; ^2^ Department of Oncology Shanghai Medical College Fudan University Shanghai China; ^3^ Department of Surgery Shanghai Pudong Hospital (Fudan University Pudong Medical Center) Shanghai China; ^4^ Department of Oncology the Affiliated Hospital of Yangzhou University Yangzhou University Yangzhou China; ^5^ Department of Molecular Diagnostics and Experimental Therapeutics Beckman Research Institute of City of Hope Comprehensive Cancer Center Duarte CA USA

**Keywords:** colorectal cancer, epithelial‐Mesenchymal Transition, immunity, prognosis, risk score model

## Abstract

Epithelial‐mesenchymal transition (EMT), a biological process involving the transformation of epithelial cells into mesenchymal cells, promotes tumour initiation and metastasis. The aim of this study was to construct an EMT molecular signature for predicting colorectal cancer (CRC) prognosis and evaluate the efficacy of the model. The risk scoring system, constructed by log‐rank test and multivariate Cox regression analysis according to EMT‐related gene expression in CRC patients from TCGA database, demonstrated the highest correlation with prognosis compared with other parameters in CRC patients. The risk scores were significantly correlated with more lymph node metastasis, distal metastasis and advanced clinical stage of CRC. The model was further successfully validated in two independent external cohorts from GEO database. Furthermore, we developed a nomogram to integrate the EMT signature with the pathological stage of CRC, which was found to perform well in predicting the overall survival. Additionally, this risk scoring model was found to be associated with immune cell infiltration, implying a potential role of EMT involved in immunity regulation in tumour microenvironment. Taken together, our novel EMT molecular model may be useful in identifying high‐risk patients who need an intensive follow‐up and more aggressive therapy, finally contributing to more precise individualized therapeutic strategies.

## 1 INTRODUCTION

Colorectal cancer (CRC) is the third most common type of cancer and the third most lethal tumour in the United States.^1^ Although advancing surgery, radiotherapy and chemotherapy have significantly prolonged the survival of CRC patients, numerous patients remain to suffer poor prognosis due to lack of sufficient therapies. Currently, prediction of CRC prognosis is still mainly dependent on tumour‐node‐metastasis (TNM) system, which ignores tumour heterogeneity and may result in over or insufficient therapies.^2^ Therefore, identification of novel and reliable prognostic biomarkers is currently an urgent task for oncologists.

During tumorigenesis, epithelial cells usually acquire unique mesenchymal cell traits to enable themselves to invade adjacent tissues before metastasizing to distant organs. This process is termed as epithelial‐mesenchymal transition (EMT), which is implicated in tumour initiation, invasion, metastasis and resistance to anti‐cancer therapies.^3^ Previous studies have attributed EMT to dysregulated inducing and transcription factors in complex CRC microenvironment, thereby driving CRC initiation and progression. For instance, inducting factors like epidermal growth F (EGF),^4^ platelet‐derived growth factor (PDGF)^5^ and fibroblast growth factor (FGF),^6^, ^7^ and transcription factors like TWIST1/2, SNAI1/2 and ZEB1 are important mediators of distal tumour metastasis in various types of cancer.^8^, ^9^, ^10^, ^11^, ^12^

Consensus molecular subtypes (CMSs), a stable classification system, are now used in CRC clinical practice. Currently, there are four CMSs available CRC patients, of which CMS4 phenotype contains various EMT‐related genes and is positively correlated with advanced TNM stage.^13^, ^14^ Despite the crucial clinical significance of EMT‐related genes in CRC, rare evidence is available for the application of their combination in more accurate prognostic prediction. Therefore, in this study, we firstly selected EMT‐associated genes that were most significantly correlated with CRC prognosis using a training cohort from TCGA database. Then, we developed a risk score model based on the selected genes and validated it in two validating cohorts from GEO database. Finally, we analysed the correlations of the risk score model with oncogenic signal activation and immune infiltration.

## 2 METHODS

### 2.1 Unsupervised clustering for EMT‐related genes

In this study, mRNA expression profile data of 613 CRC tissues were retrieved from the TCGA database which is available online at https://portal.gdc.cancer.gov/. Details of samples are presented in Table 1. On the other hand, 1184 EMT‐associated genes were retrieved from the dbEMT database which is available online at. http://dbemt.bioinfo‐minzhao.org/.^15^ The retrieved genes are shown in Supplemental Table S1. Out of the 1184 retrieved genes, 1169 EMT genes were detected in TCGA‐derived mRNA expression profiles of CRC tissues. An unsupervised clustering method was used to detect and categorize unique EMT phenotypes on 1169 EMT gene expression profiles for analysis. The consensus clustering algorithm was used to determine cluster numbers, and the procedure was repeated 50 times to ensure stability of cluster classification, using the ConsensuClusterPlus package.

**TABLE 1:** Clinical characteristic of 613 CRC tissues in TCGA database

Characteristic	No. of Patients
Age	
>60	421
<=60	192
Stage	
Stage I	103
Stage II	227
Stage III	177
Stage IV	86
Not available	20
PT	
T1	19
T2	104
T3	418
T4	69
Tis	1
PN	
T0	348
T1	146
NX	2
Not available	2
PM	
M0	452
M1	85
MX	66
Not available	10
Sex	
Female	287
Male	326
Status	
Alive	485
Dead	128

### 2.2 Identification of differentially expressed genes (DEGs) in EMT distinct phenotypes

Limma R package was used to identify differentially expressed genes in two phenotype patterns. In this study, a threshold value of |log2FC| > 1 and *P* <.05 was set as significance criteria. Afterwards, R package clusterProfiler was applied to perform Gene Ontology (GO) and Kyoto Encyclopedia of Genes and Genomes (KEGG) enrichment analyses to identify significantly enriched terms.

### 2.3 Construction and evaluation of an EMT molecular signature for CRC prognosis

In this study, EMT‐associated genes that were correlated with OS significantly (*P* < .05) were selected using the Kaplan‐Meier analysis. Thereafter, the selected genes were subjected to multivariate Cox regression analysis to determine their weights for prediction of CRC prognosis. An EMT molecular signature was constructed from the risk scoring system based on gene expression profile and the determined weight values. The median risk scores were considered as cut‐off values for distinguishing between high‐risk and low‐risk groups. The predicting performance of the constructed EMT molecular signature was evaluated using Kaplan‐Meier and receiver operating characteristic curve (ROC) analyses. The univariate and multivariate analysis based on Cox regression model was used to evaluate the prognostic values of EMT risk scores and clinical characteristics. Function difference in different subgroups of CRC was analysed through Gene Set Enrichment Analysis (GSEA).

### 2.4 Validation of the constructed EMT molecular signature for predicting CRC prognosis

Two independent CRC cohorts were retrieved from the GEO database (NO. GSE14333; GSE17538), which are available online at https://www.ncbi.nlm.nih.gov/geo/., and applied to validate the efficacy of the constructed EMT molecular signature.

### 2.5 Evaluation of immune infiltrating cell proportion

The proportion of 22 human haematopoietic cell phenotypes in CRC tissues were evaluated using CIBERSORT method and leucocyte characteristic gene array (LM22). CIBERSORT (https://cibersort.stanford.edu/) technique profiles the composition of cells based on gene expression levels. On the other hand, LM22 utilizes 613 genes to detect 22 categories of immune cells containing myeloid subpopulation, natural killer (NK) cell, plasmocyte, naive and memory B cell, and seven T cell phenotypes in a given sample. For each sample, the sum of the proportions of the evaluated immune infiltrating cells is equal to 1.

### 2.6 Statistical analysis

Statistical analysis was performed using R (version 3.4.1; http//www.rproject.org/) and MedCalc version 16.1. (MedCalc Software) software. Unpaired t test was performed to determine DEGs between two groups in public data sets at α_0.05_. Kaplan‐Meier model was used to plot survival curves, and intergroup difference was compared using log‐rank test. Multivariate Cox regression analysis was performed on EMT genes to build a combined prognostic signature, where the calculated median values were used to stratify patient OS. The univariate and multivariate analysis based on Cox proportional hazards regression model was performed to detect significantly independent prognostic factors for OS. A two‐sided *P* value less than .05 was considered significantly different.

## 3 RESULTS

### 3.1 Expression of EMT‐related genes in tumours of patients with different subtypes of CRC

**FIGURE 1:**
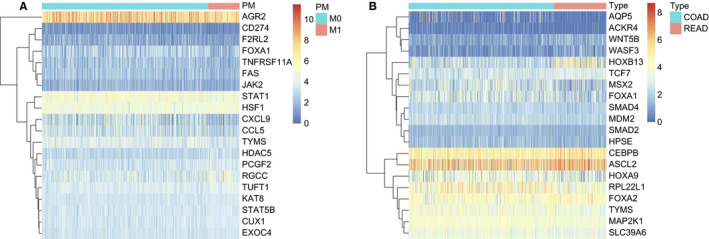
Expression patterns of EMT‐related genes in colorectal cancer. A, The expression levels of top 20 EMT‐related genes in colorectal cancer with metastasis or non‐metastasis. B, The expression levels of top 20 EMT‐related genes in colon cancer or rectal cancer

A total of 224 differentially expressed EMT‐associated genes were identified in CRC with and without distal metastasis. Figure 1A showed differential gene expression profile of 20 highly expressed genes from a pool of 224 differentially expressed genes. Afterwards, the differentially expressed genes between colon cancer and rectal cancer were selected. The heatmap demonstrated the top 20 differential genes of total 266 differentially expressed genes (Figure 1B). Further investigation revealed FOXA1 and TYMS mRNA expression were significantly associated with the clinical stage of CRC (*P* < .05 for stage III/IV vs. stage I/II; Supplemental Figure S1A‐F).

### 3.2 EMT expression patterns mediated by EMT‐associated genes and their functional annotation

**FIGURE 2:**
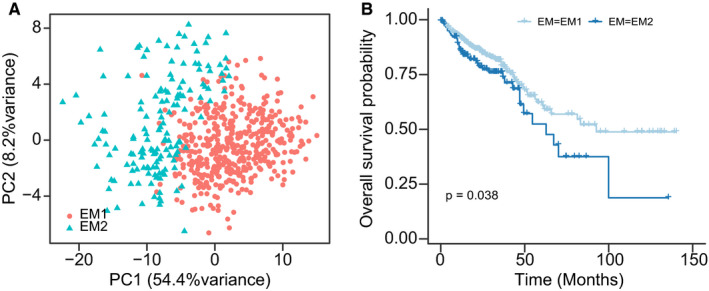
Prognostic stratification of the TCGA cohorts by EMT‐related genes. A, Principal component analysis (PCA) of EM1/2 subgroups. B, Kaplan‐Meier model shows the OS of the TCGA cohorts is stratified by EM1/EM2 expression pattern

In this study, we divided CRC patients into two distinct molecular patterns based on the EMT‐related gene expression using unsupervised clustering through ConsensusClusterPlus R package. The first pattern (EM1) was identified in 461 CRC cases, while the second pattern (EM2) was identified in 152 CRC cases (Supplemental Figure S2A‐C). Principal component analysis further revealed two completely distinct EMT modification patterns, in EM1 and EM2 gene transcription profiles (Figure 2A). EM2 subgroup, characterized by more distal metastasis and advanced clinical stage, showed a significantly poorer overall survival compared to EM1 subgroup (Figure 2B; Supplemental Figure S3).

**FIGURE 3:**
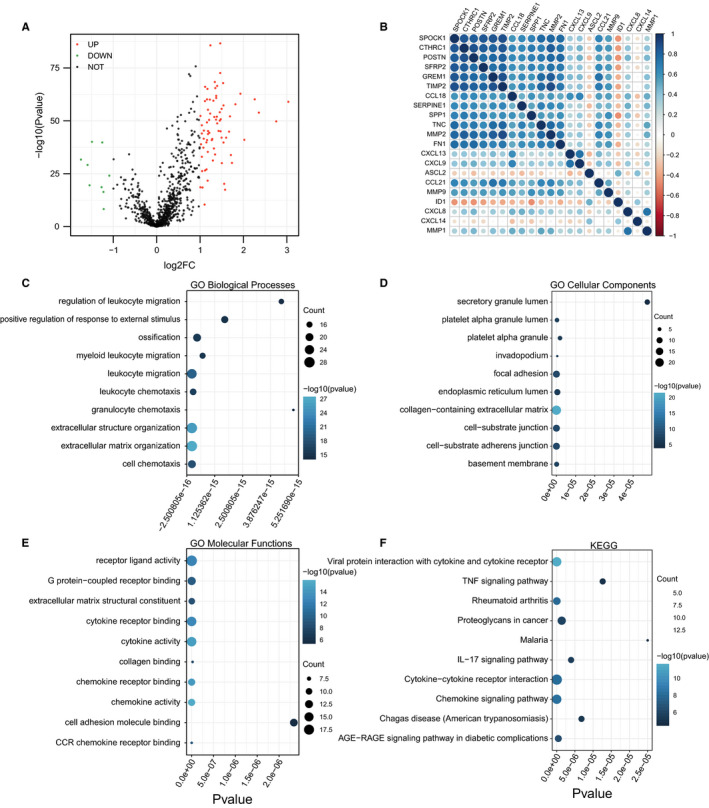
Correlations of EMT‐related genes and functional annotation of differentially expressed genes (DEGs) in EM1/2 subgroups. A, Volcano plots showing differentially expressed genes. B, The correlations of the 21 EMT‐related genes with each other. C, Biological process analysis of DEGs in EM1/2 subgroups. D, Cellular component analysis of DEGs in EM1/2 subgroups. E, Molecular function analysis of DEGs in EM1/2 subgroups. F, KEGG pathway analysis of DEGs in EM1/2 subgroups

Analysis of the 82 DEGs showed that 73 genes were highly expressed in the EM1, while 9 genes were highly expressed in EM2 (Figure 3A). Moreover, 21 DEGs had a fold change of more than 1.5 and were found to be significantly correlated with each other (Figure 3B). Analysis of the biological behaviours of the 82 DEGs using GO and KEGG tools showed that they were associated with cell adhesion, chemokine and cell junction, which play a significant role in the distal metastasis of CRC (Figure 3C‐F).

### 3.3 Development of an EMT molecular signature from the training set

**FIGURE 4:**
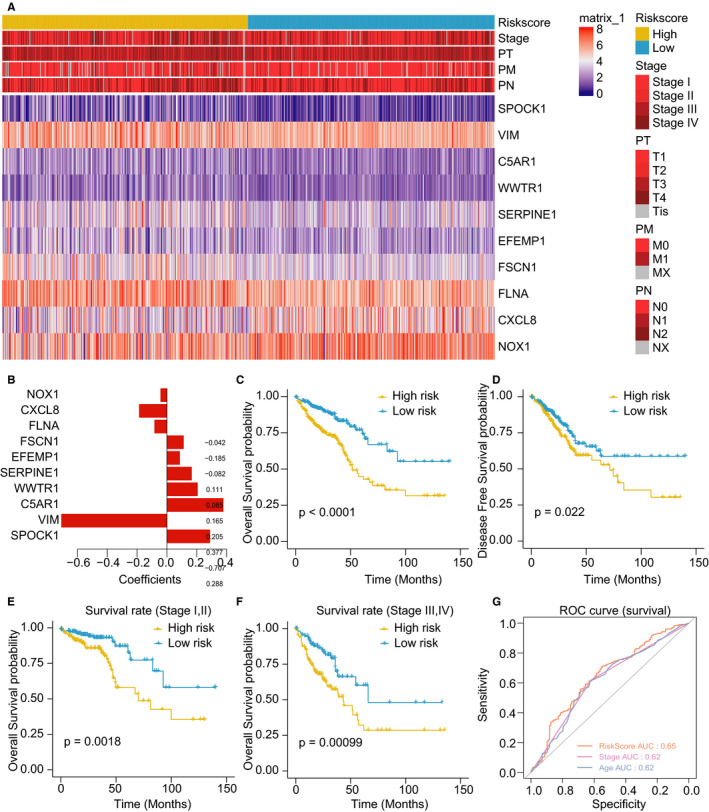
Construction of a prognostic risk score model dependent on EMT‐related genes. A, The heatmap shows the expression of 10 EMT‐related genes in low‐ and high‐risk colorectal cancer patients. B, The coefficients of 10 EMT‐related prognostic genes calculated by multivariate Cox regression analysis. C, D, The Kaplan‐Meier model shows overall survival (OS) (C) and disease‐free survival (DFS) (D) in the TCGA cohort. E, F, OS curves in TCGA stage I‐II colorectal cancer patients (E) and TCGA stage III‐IV patients (F). G, ROC analysis evaluating the survival predictive performance of the risk score model and clinicopathological characteristics

Log‐rank test identified 10 DEGs that were associated with CRC prognosis, and their correlations with CRC features were shown in Figure 4A. In addition, 10 DEGs were identified as predictors of OS of CRC patients through stepwise multivariate Cox regression analysis (Figure 4B) and further utilized for constructing a prognostic risk model (risk score = SPOCK1 × 0.287634‐VIM × ‐0.70744 + C5AR1 × 0.376991 + WWTR1 × 0.204543 + SERPINE1 × 0.164739 + EFEMP1 × 0.085466 + FSCN1 × 0.11085‐FLNA × ‐0.08217‐CXCL8 × 0.18518‐NOX1 × 0.04164). The gene expression risk score was calculated using the TCGA cohorts, and the median risk score was defined as the cut‐off value for dichotomization. The survival analysis indicated CRC patients with high‐risk scores had a significantly shorter OS and DFS as compared with those in the low‐risk group (*P* < .05; Figure 4C,D). Moreover, low‐risk patients were found to have a significantly better OS at than the high‐risk ones in both stage I‐II and stage III‐IV (Figure 4E,F). The results further showed that the risk scores were significantly correlated with more lymph node metastasis (N2 vs. N0 and N2 vs. N1), distal metastasis (M1 *vs*. M0) and advanced clinical stage (stage II/III/IV vs. stage I) of CRC (Supplemental Figure S4A‐D). However, no significant correlations of risk scores with sex or age were found (*P* > .05; Supplemental Figure S4E‐F). Besides, the ROC analysis showed that the constructed risk score model performed better than TNM stage or age in predicting patient survival (area under the curve [AUC] = 0.65 for risk score model *vs*. 0.62 for TNM stage and age, Figure 4G). Finally, patients with EM2 phenotype had a remarkably higher risk score than those with EM1 phenotype (Supplemental Figure S4G). The ROC analysis demonstrated that the risk model score could significantly stratify the EM phenotype with AUC of 0.72 (Supplemental Figure S4H).

### 3.4 Validation of the EMT signature in the external cohorts

**FIGURE 5:**
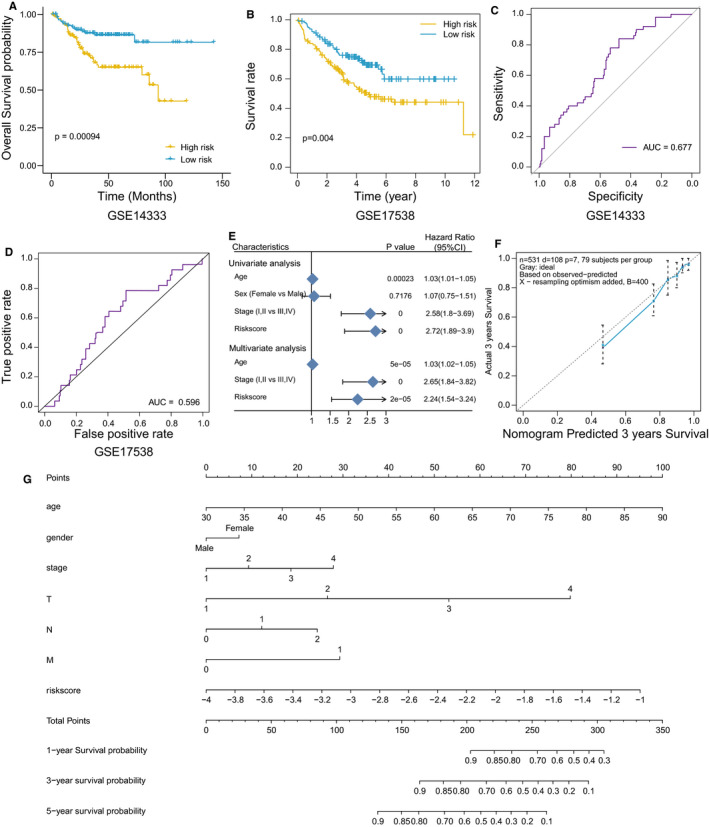
The predictive value of risk score model in the prognosis of GEO and TCGA cohorts. A, B, Kaplan‐Meier plots of overall survival (OS) in GSE14333 (A) and GSE17538 (B) data sets. C, D, ROC analysis for the OS predicting performance of the risk score model in GEO14333 (C) and GEO17538 (D). E, The prognostic value of the risk score model and clinicopathological factors in TCGA cohort analysed by univariate or multivariate Cox regression analyses. F, Nomogram predicting OS of colorectal cancer patients from TCGA cohort. G, Calibration plot of the nomogram for predicting the probability of 3‐year OS

Two external cohort cohorts (GSE14333, n = 226 and GSE17538, n = 244) were used to validate the stability and the reliability of the EMT risk score model in predicting OS of CRC patients. The results showed that the high‐risk group patients had a significantly poorer prognosis compared with the patients in the low‐risk group in both cohorts (Figure 5A,B). In addition, the ROC analysis was further performed to evaluate the prognostic accuracy of the risk score model, demonstrating an AUC of 0.677 for GSE14333 (Figure 5C) and an AUC of 0.596 for GSE17538 (Figure 5D).

### 3.5 Independent prognostic value of the EMT signature

A univariate Cox regression model showed the prognostic values of age (HR: 1.03, 95% CI: 1.01‐1.05; *P* < .001), clinical stage (HR: 2.58, 95% CI: 1.80‐3.69; *P* < .001) and risk score (HR: 2.72, 95% CI: 1.89‐3.90; *P* < .001) in CRC patients. Multivariate Cox regression model analysis further exhibited the risk score could serve as an independent prognostic predictor for the OS of CRC patients (HR: 2.24, 95% CI: 1.54‐3.24; *P* < .001; Figure 5E).

### 3.6 Development of a nomogram for predicting OS in CRC

A nomogram, which integrated the EMT risk score model and the pathological stage, was constructed based on the TCGA cohort to predict the OS of CRC patients (Figure 5F). The calibration curves for 3‐year OS estimates revealed an acceptable model calibration, with excellent correlation between the OS estimates from the nomogram, and the actual outcome of the TCGA data set (Figure 5G).

### 3.7 Signal transduction pathways and immune cell infiltration in CRC patients stratified by the EMT signature

**FIGURE 6:**
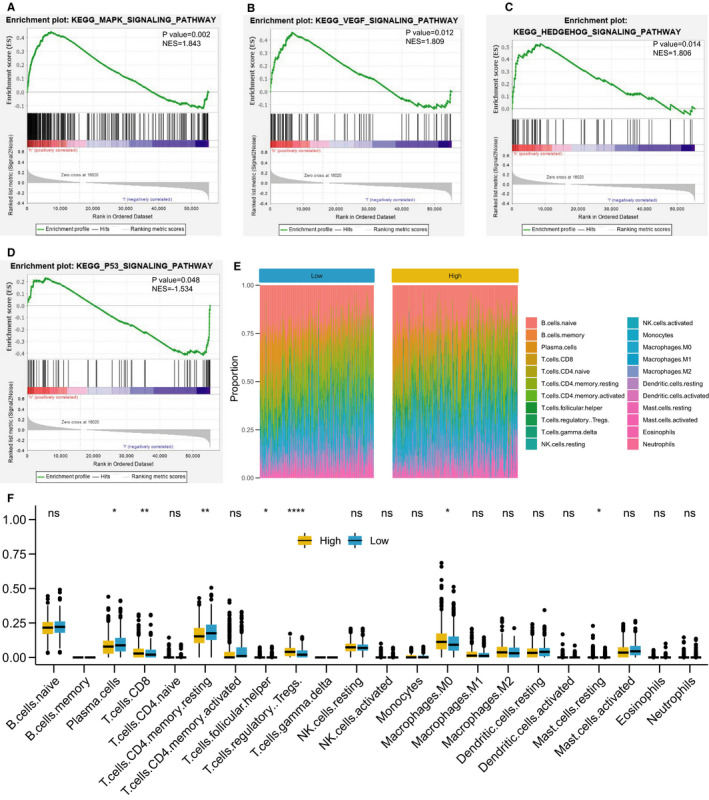
Signalling pathway analysis and immune cell infiltration in high‐ and low‐risk group. A‐C, Activated MAPK (A), VEGF (B) and HEDGEHOG (C) signalling pathway in the high‐risk group. D, Activated p53 signalling pathway in the low‐risk group. E, The proportions of infiltrated immune cells in high‐ and low‐risk score group. F, Comparison of the proportions of infiltrated immune cells between high‐ and low‐risk score group

As shown in Figure 6A‐D, GSEA analysis suggested oncogenic MAPK, VEGF and HEDGEHOG signal pathway were activated in high‐risk group, while anti‐cancer P53 signal pathway was activated in low‐risk group, which may partially explain the prognosis difference between high‐ and low‐risk groups. CIBERSORT, a deconvolution algorithm to analyse the infiltration immune cell type in tumours, was applied to compare the differences of infiltrated immune cells between low‐risk and high‐risk group (Figure 6E). As shown in Figure 6F, we found the proportions of CD8(+) T cells, T regulatory cells and M0 macrophage cells were significantly higher in the high‐risk group than the low‐risk group, which was opposite for plasma cells, CD4(+) memory resting T cells and follicular T helper cells. These results suggested the EMT was involved in immune regulation in tumour microenvironment.

## 4 DISCUSSION

Nowadays, traditional prognostic prediction is mainly dependent on American Joint Committee on Cancer (AJCC) TNM classification. However, a large proportion of patients with same clinicopathological features are reported to have entirely different clinical outcome after receiving uniform therapeutic regimes.^16^ This dilemma may reflect high tumoural heterogeneity among individuals.^17^ Therefore, we extremely need a novel prognostic biomarker in combination with current TMM staging system to improve the prognostic evaluation and clinical management of CRC patients. EMT, a well‐known molecular event driving CRC progression, has been regarded as a main orchestrator of intratumoural heterogeneity.^18^, ^19^ Accumulating evidence demonstrates that EMT plays a crucial role in tumour metastasis and chemotherapy resistance.^20^, ^21^ In this study, we systematically analysed EMT‐associated gene expression profiles in CRC tissues and constructed a risk score model based on EMT‐related prognostic genes, which may serve as a reliable tool for the individualized evaluation of CRC prognosis in clinical practice.

In this study, we firstly divided CRC patients into EM1 and EM2 subgroup through consensus clustering algorithm according to EMT‐related genes in CRC tissues, of which EM2 subgroup patients showed a poorer survival compared to EM1 subgroup. GO analysis and KEGG analysis exhibited the survival difference was mainly associated with cell adhesion, chemokine and cell junction. These biological functions are also closely linked to the invasion and dissemination of CRC cells, which is identified as the leading unfavourable factor affecting patient survival.

Afterwards, a prognostic risk factor model, which classifies CRC patients into low‐ and high‐risk categories in OS and DFS, was constructed according to ten selected EMT‐related prognostic genes (SPOCK1, VIM, C5AR1, WWTR1, SERPINE1, EFEMP1, FSCN1, FLNA, CXCL8, NOX1). Some of these genes have been reported to be involved in the development and progression of CRC. FLNA ser2152 phosphorylated by c‐Met‐AKT enhanced c‐Met promoter activity by its interaction with smad2 to induce epithelial‐mesenchymal transition, promoting chemoresistance of CRC.^22^ c‐Met expression in CRC samples was not only significantly correlated with advanced AJCC staging, but could be integrated with the expression of receptor proteins including EGFR, FAK and CD44v6 for prognosis stratification.^23^ Fascin actin‐bundling protein 1 (FSCN1) is involved in the regulation of YAP1‐induced CRC proliferation and metastasis.^24^ SPOCK1 is associated with tumour size and TNM stage, promotes the epithelial‐mesenchymal transition (EMT) and activates PI3K/Akt signalling pathway to enhance the proliferative and invasive ability of CRC cells.^25^, ^26^ Except for CRC, some of these genes have also been found to be associated with the progression of other cancers. For instance, SERPINE1, a critical target of the TP53/miR‐34a axis affecting the malignant characteristics of pancreatic ductal adenocarcinoma (PDAC), may become a potential biomarker for early detection.^27^ Upon liver injury, NOX1 gene of liver macrophages was activated by damage‐associated molecular patterns secreted by dying hepatocytes to accelerate the development of hepatoma.^28^ RNAi screen identified the potential oncogenic role of SPOCK1 in human brain metastasis‐initiating cells.^29^ Both in the training set and validation sets, we found the constructed risk model could effectively stratify the OS of CRC patients. In addition, a nomogram that integrated our risk model and the pathological stage was developed to predict the OS of CRC patients based on the TCGA CRC set. These findings strongly suggested this ten‐gene EMT signature would be helpful for identifying the high‐risk subpopulation from patients within the same clinicopathological features and therefore benefiting the individualized follow‐up protocol and therapy decision. Our findings are in line with a previous study in glioma suggesting the reliability and stability of the prognostic risk factor model based on EMT‐related genes.^30^

The signal pathway activated in the high‐risk group, including MAPK, VEGF and HEDGEHOG signal pathway, which are all associated with tumour malignancy, may explain the reasons for adverse prognosis. Furthermore, the two patterns had significantly distinct immune cell infiltration characterization, implying a potential link between EMT phenotype and tumour immunity. Previous studies have showed EMT‐related genes included in our signature were involved in the regulation of immunity infiltration in TME. For instance, CXCL8, mainly releasing from macrophages, up‐regulates PD‐L1^+^ expression of macrophages, exacerbating the immunosuppressive status of gastric microenvironment.^31^ Therapeutic inhibition of C5AR1 in combination with chemotherapy reprogrammed immunosuppressive microenvironments, resulting in CD8^+^ T cell–dependent antitumour immune responses associated with decreased local and peripheral T cell receptor β diversity.^32^ The neutrophils have been demonstrated to promote carcinogenesis in various ways, including releasing neutrophil extracellular traps to promote tumour metastasis^33^ and enhancing the malignant potential of circulating tumour cells (CTCs).^34^ The recruitment of neutrophils into human hepatocellular carcinoma (HCC) is mainly regulated by CXCL8.^35^ WWTR1 (also known as TAZ) and YAP1 as the major downstream effectors of the Hippo pathway are found not only to trigger numerous cell‐autonomous responses, but also to participate in choreographing tumour‐stromal interactions.^36^

In conclusion, we constructed a risk scoring system based on 10 EMT‐related genes, which potentially benefits the precise prediction for CRC prognosis. In future, multi‐centre validations based on sufficient clinical resources are necessary for confirming the potential utility of our signature. In addition, mechanism investigations should be made to further clarify the correlation between EMT phenotype and tumour immunity in CRC development.

## CONFLICT OF INTEREST

None of the authors has any potential conflicts to disclose.

## AUTHOR CONTRIBUTION

**Zezhi Shan:** Conceptualization (lead); Data curation (lead); Formal analysis (lead); Methodology (lead); Resources (lead); Software (lead); Validation (lead); Writing‐original draft (lead). **Wen Wu:** Formal analysis (equal); Methodology (equal). **Xuebing Yan:** Data curation (equal); Formal analysis (equal); Methodology (equal); Writing‐original draft (equal). **Yongzhi Yang:** Conceptualization (equal); Data curation (equal); Formal analysis (equal); Investigation (equal); Methodology (equal); Project administration (equal); Supervision (lead); Writing‐original draft (equal). **Dakui Luo:** Data curation (equal); Methodology (equal); Visualization (equal). **Qi Liu:** Data curation (equal); Formal analysis (equal); Methodology (equal); Resources (equal); Software (equal); Visualization (equal). **Xinxiang Li:** Conceptualization (equal); Investigation (equal); Project administration (equal); Writing‐review & editing (equal). **Ajay Goel:** Conceptualization (equal); Investigation (equal); Supervision (equal); Visualization (equal); Writing‐review & editing (equal). **Yanlei Ma:** Conceptualization (lead); Funding acquisition (lead); Investigation (lead); Writing‐review & editing (lead).

## Supporting information

Fig S1

Fig S2

Fig S3

Fig S4

Table S1

## Data Availability

We obtained the clinicopathological data sets from TCGA (https://portal.gdc.cancer.gov/) and GEO (https://www.ncbi.nlm.nih.gov/geo/). A total of 1184 EMT‐related genes were obtained from dbEMT database (http://dbemt.bioinfo‐minzhao.org/).

## References

[1] Siegel RL , Miller KD , Jemal A . Cancer statistics, 2019. CA Cancer J Clin. 2019;69(1):7‐34.https://doi.org10.3322/caac.21551. 30620402 10.3322/caac.21551

[2] Weiser MR , Gönen M , Chou JF , Kattan MW , Schrag D . Predicting survival after curative colectomy for cancer: individualizing colon cancer staging. J Clin Oncol. 2011;29(36):4796‐4802.https://doi.org10.1200/JCO.2011.36.5080. 22084366 10.1200/JCO.2011.36.5080 PMC3664036

[3] Yang J , Antin P , Berx G , et al. Guidelines and definitions for research on epithelial‐mesenchymal transition. Nat Rev Mol Cell Biol. 2020;21(6):341‐352.https://doi.org10.1038/s41580‐020‐0237‐9. 32300252 10.1038/s41580-020-0237-9 PMC7250738

[4] Kim J , Kong J , Chang H , Kim H , Kim A . EGF induces epithelial‐mesenchymal transition through phospho‐Smad2/3‐Snail signaling pathway in breast cancer cells. Oncotarget. 2016;7(51):85021‐85032.https://doi.org10.18632/oncotarget.13116. 27829223 10.18632/oncotarget.13116 PMC5356716

[5] Tam WL , Lu H , Buikhuisen J , et al. Protein kinase C α is a central signaling node and therapeutic target for breast cancer stem cells. Cancer Cell. 2013;24(3):347‐364.https://doi.org10.1016/j.ccr.2013.08.005. 24029232 10.1016/j.ccr.2013.08.005 PMC4001722

[6] Shirakihara T , Horiguchi K , Miyazawa K , et al. TGF‐β regulates isoform switching of FGF receptors and epithelial‐mesenchymal transition. EMBO J. 2011;30(4):783‐795.https://doi.org10.1038/emboj.2010.351. 21224849 10.1038/emboj.2010.351 PMC3041949

[7] Miyazono K , Ehata S , Koinuma D . Tumor‐promoting functions of transforming growth factor‐β in progression of cancer. Ups J Med Sci. 2012;117(2):143‐152.https://doi.org10.3109/03009734.2011.638729. 22111550 10.3109/03009734.2011.638729 PMC3339546

[8] Dongre A , Weinberg RA . New insights into the mechanisms of epithelial‐mesenchymal transition and implications for cancer. Nat Rev Mol Cell Biol. 2019;20(2):69‐84.https://doi.org10.1038/s41580‐018‐0080‐4. 30459476 10.1038/s41580-018-0080-4

[9] Xu Y , Lee D‐K , Feng Z , et al. Breast tumor cell‐specific knockout of inhibits cancer cell plasticity, dissemination, and lung metastasis in mice. Proc Natl Acad Sci USA. 2017;114(43):11494‐11499.https://doi.org10.1073/pnas.1618091114. 29073077 10.1073/pnas.1618091114 PMC5664488

[10] Krebs AM , Mitschke J , Lasierra Losada M , et al. The EMT‐activator Zeb1 is a key factor for cell plasticity and promotes metastasis in pancreatic cancer. Nat Cell Biol. 2017;19(5):518‐529.https://doi.org10.1038/ncb3513. 28414315 10.1038/ncb3513

[11] Zheng X , Carstens JL , Kim J , et al. Epithelial‐to‐mesenchymal transition is dispensable for metastasis but induces chemoresistance in pancreatic cancer. Nature. 2015;527(7579):525‐530.https://doi.org10.1038/nature16064. 26560028 10.1038/nature16064 PMC4849281

[12] Tran HD , Luitel K , Kim M , Zhang K , Longmore GD , Tran DD . Transient SNAIL1 expression is necessary for metastatic competence in breast cancer. Can Res. 2014;74(21):6330‐6340.https://doi.org10.1158/0008‐5472.CAN‐14‐0923. 10.1158/0008-5472.CAN-14-0923 PMC4925010 25164016

[13] Guinney J , Dienstmann R , Wang X , et al. The consensus molecular subtypes of colorectal cancer. Nat Med. 2015;21(11):1350‐1356.https://doi.org10.1038/nm.3967. 26457759 10.1038/nm.3967 PMC4636487

[14] Lenz H‐J , Ou F‐S , Venook AP , et al. Impact of Consensus Molecular Subtype on Survival in Patients With Metastatic Colorectal Cancer: Results From CALGB/SWOG 80405 (Alliance). J Clin Oncol. 2019;37(22):1876‐1885.https://doi.org10.1200/JCO.18.02258. 31042420 10.1200/JCO.18.02258 PMC6675593

[15] Zhao M , Liu Y , Zheng C , Qu H . dbEMT 2.0: An updated database for epithelial‐mesenchymal transition genes with experimentally verified information and precalculated regulation information for cancer metastasis. J Genet Genomics. 2019;46(12):595‐597.https://doi.org10.1016/j.jgg.2019.11.010. 31941584 10.1016/j.jgg.2019.11.010

[16] Bathe OF , Farshidfar F . From genotype to functional phenotype: unraveling the metabolomic features of colorectal cancer. Genes (Basel). 2014;5(3):536‐560.https://doi.org10.3390/genes5030536. 25055199 10.3390/genes5030536 PMC4198916

[17] Bjerkvig R , Tysnes BB , Aboody KS , Najbauer J , Terzis AJA . Opinion: the origin of the cancer stem cell: current controversies and new insights. Nat Rev Cancer. 2005;5(11):899‐904. 16327766 10.1038/nrc1740

[18] Mani SA , Guo W , Liao M‐J , et al. The epithelial‐mesenchymal transition generates cells with properties of stem cells. Cell. 2008;133(4):704‐715.https://doi.org10.1016/j.cell.2008.03.027. 18485877 10.1016/j.cell.2008.03.027 PMC2728032

[19] Pang R , Law WL , Chu ACY , et al. A subpopulation of CD26+ cancer stem cells with metastatic capacity in human colorectal cancer. Cell Stem Cell. 2010;6(6):603‐615.https://doi.org10.1016/j.stem.2010.04.001. 20569697 10.1016/j.stem.2010.04.001

[20] Williams ED , Gao D , Redfern A , Thompson EW . Controversies around epithelial‐mesenchymal plasticity in cancer metastasis. Nat Rev Cancer. 2019;19(12):716‐732.https://doi.org10.1038/s41568‐019‐0213‐x. 31666716 10.1038/s41568-019-0213-x PMC7055151

[21] Shibue T , Weinberg RA . EMT, CSCs, and drug resistance: the mechanistic link and clinical implications. Nat Rev Clin Oncol. 2017;14(10):611‐629.https://doi.org10.1038/nrclinonc.2017.44. 28397828 10.1038/nrclinonc.2017.44 PMC5720366

[22] Cheng M , Jiang Y , Yang H , Zhao D , Li L , Liu X . FLNA promotes chemoresistance of colorectal cancer through inducing epithelial‐mesenchymal transition and smad2 signaling pathway. Am J Cancer Res. 2020;10(2):403‐423. 32195017 PMC7061762

[23] Garouniatis A , Zizi‐Sermpetzoglou A , Rizos S , Kostakis A , Nikiteas N , Papavassiliou AGFAK . CD44v6, c‐Met and EGFR in colorectal cancer parameters: tumour progression, metastasis, patient survival and receptor crosstalk. Int J Colorectal Dis. 2013;28(1):9‐18.https://doi.org10.1007/s00384‐012‐1520‐9. 22733437 10.1007/s00384-012-1520-9

[24] Ou C , Sun Z , He X , et al. Targeting YAP1/LINC00152/FSCN1 Signaling Axis Prevents the Progression of Colorectal Cancer. Adv Sci (Weinh). 2020;7(3):1901380. https://doi.org10.1002/advs.201901380. 32042551 10.1002/advs.201901380 PMC7001651

[25] Zhao P , Guan H‐T , Dai Z‐J , Ma Y‐G , Liu X‐X , Wang X‐J . Knockdown of SPOCK1 Inhibits the Proliferation and Invasion in Colorectal Cancer Cells by Suppressing the PI3K/Akt Pathway. Oncol Res. 2016;24(6):437‐445.https://doi.org10.3727/096504016X14685034103554. 28281964 10.3727/096504016X14685034103554 PMC7838686

[26] Zhang J , Zhi X , Shi S , et al. SPOCK1 is up‐regulated and promotes tumor growth via the PI3K/AKT signaling pathway in colorectal cancer. Biochem Biophys Res Comm. 2017;482(4):870‐876.https://doi.org10.1016/j.bbrc.2016.11.126. 27889608 10.1016/j.bbrc.2016.11.126

[27] Akula SM , Ruvolo PP , McCubrey JA . TP53/miR‐34a‐associated signaling targets expression in human pancreatic cancer. Aging (Albany NY). 2020;12(3):2777‐2797.https://doi.org10.18632/aging.102776. 31986125 10.18632/aging.102776 PMC7041729

[28] Liang S , Ma H‐Y , Zhong Z , et al. NADPH Oxidase 1 in Liver Macrophages Promotes Inflammation and Tumor Development in Mice. Gastroenterology. 2019;156(4):https://doi.org10.1053/j.gastro.2018.11.019. 10.1053/j.gastro.2018.11.019 PMC6409207 30445007

[29] Singh M , Venugopal C , Tokar T , et al. RNAi screen identifies essential regulators of human brain metastasis‐initiating cells. Acta Neuropathol. 2017;134(6):923‐940.https://doi.org10.1007/s00401‐017‐1757‐z. 28766011 10.1007/s00401-017-1757-z

[30] Tao C , Huang K , Shi J , Hu Q , Li K , Zhu X . Genomics and prognosis analysis of epithelial‐mesenchymal transition in glioma. Front Oncol. 2020;10:183. https://doi.org10.3389/fonc.2020.00183. 32154177 10.3389/fonc.2020.00183 PMC7047417

[31] Lin C , He H , Liu H , et al. Tumour‐associated macrophages‐derived CXCL8 determines immune evasion through autonomous PD‐L1 expression in gastric cancer. Gut. 2019;68(10):1764‐1773.https://doi.org10.1136/gutjnl‐2018‐316324. 30661053 10.1136/gutjnl-2018-316324

[32] Medler TR , Murugan D , Horton W , et al. Complement C5a fosters squamous carcinogenesis and limits T cell response to chemotherapy. Cancer Cell. 2018;34(4):561‐578.e6.https://doi.org10.1016/j.ccell.2018.09.003. 30300579 10.1016/j.ccell.2018.09.003 PMC6246036

[33] Yang L , Liu Q , Zhang X , et al. DNA of neutrophil extracellular traps promotes cancer metastasis via CCDC25. Nature. 2020;583(7814):133‐138.https://doi.org10.1038/s41586‐020‐2394‐6. 32528174 10.1038/s41586-020-2394-6

[34] Szczerba BM , Castro‐Giner F , Vetter M , et al. Neutrophils escort circulating tumour cells to enable cell cycle progression. (1476–4687 (Electronic)). 10.1038/s41586-019-0915-y 30728496

[35] Peng Z‐P , Jiang Z‐Z , Guo H‐F , et al. Glycolytic activation of monocytes regulates the accumulation and function of neutrophils in human hepatocellular carcinoma. J Hepatol. 2020;73(4):906‐917.https://doi.org10.1016/j.jhep.2020.05.004. 32407813 10.1016/j.jhep.2020.05.004

[36] Zanconato F , Cordenonsi M , Piccolo S . YAP and TAZ: a signalling hub of the tumour microenvironment. Nat Rev Cancer. 2019;19(8):454‐464.https://doi.org10.1038/s41568‐019‐0168‐y. 31270418 10.1038/s41568-019-0168-y

