# Supplementary figures and images for: A novel epithelial‐mesenchymal transition molecular signature predicts the oncological outcomes in colorectal cancer
Source: J Cell Mol Med. 2021 Mar 4;25(7):3194–204. doi: 10.1111/jcmm.16387 (PMC8034457; doi:10.1111/jcmm.16387)

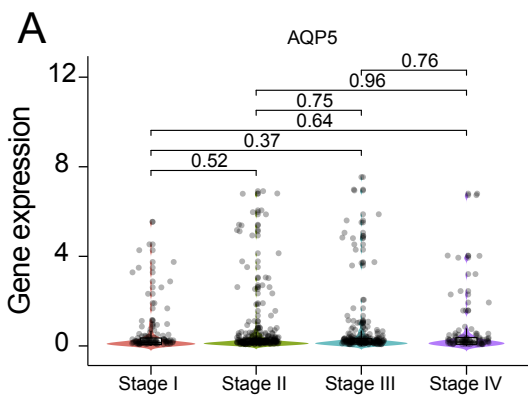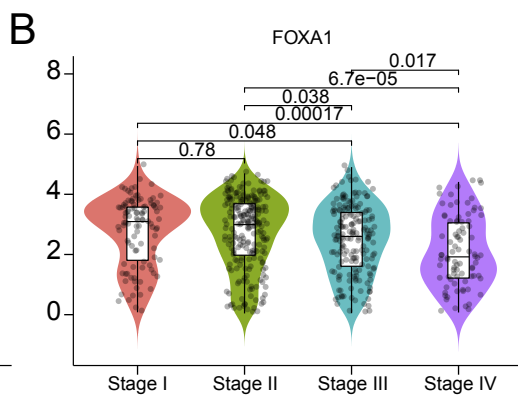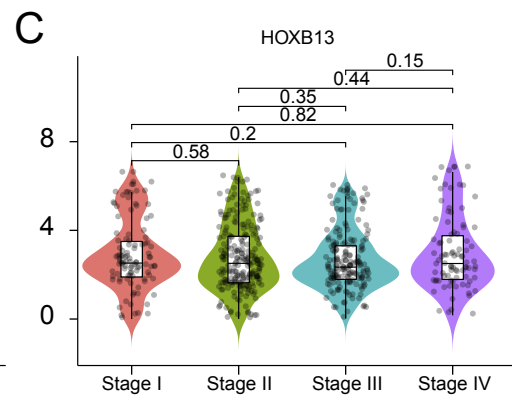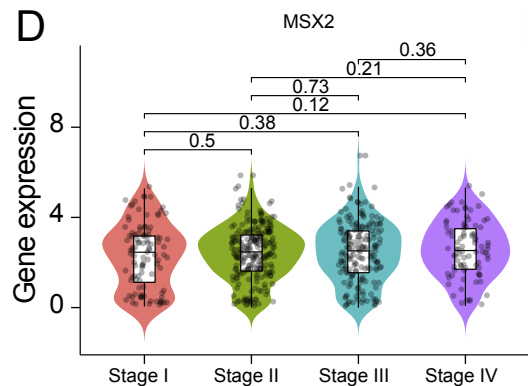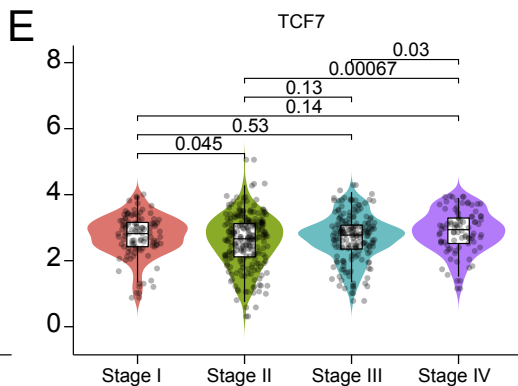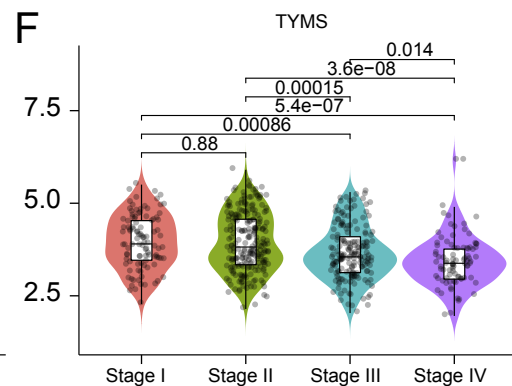

Supplement: Supplementary file 1 — Fig S1 [file JCMM-25-3194-s003.pdf]

A

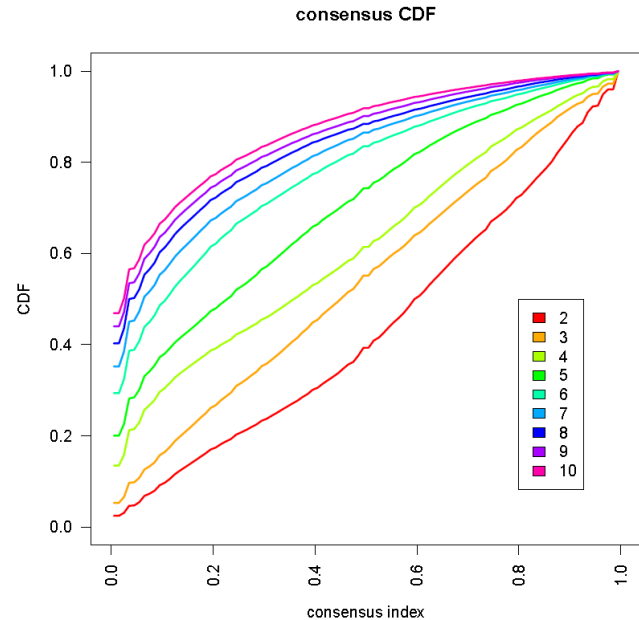

B

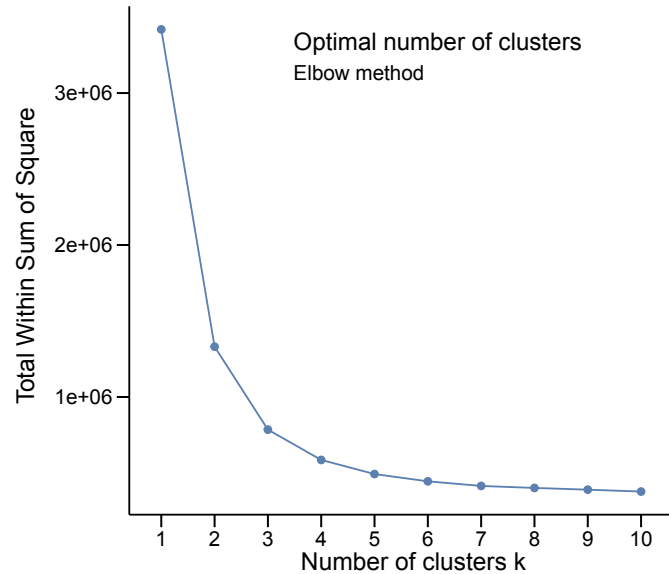

C

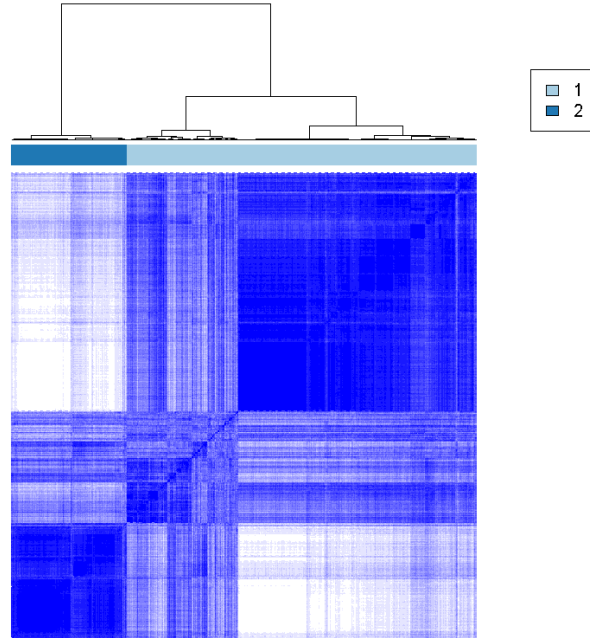

Supplement: Supplementary file 2 — Fig S2 [file JCMM-25-3194-s001.pdf]

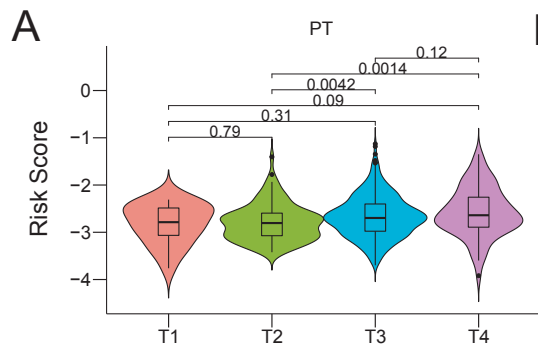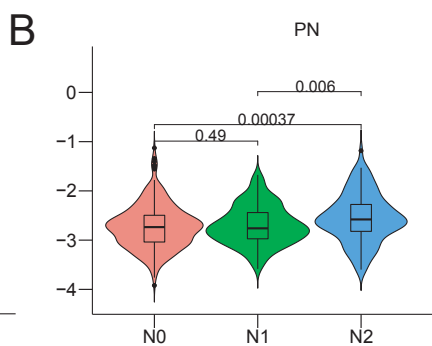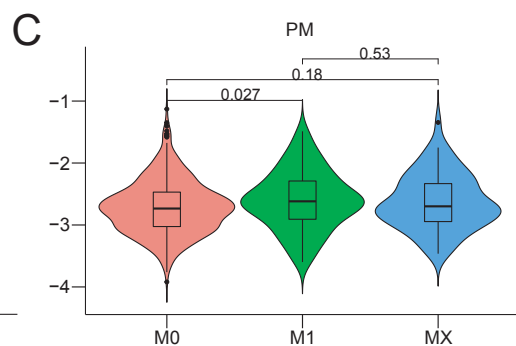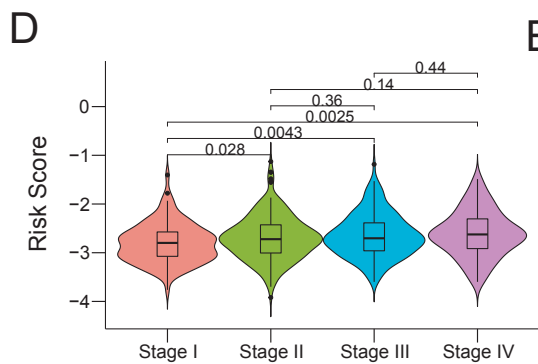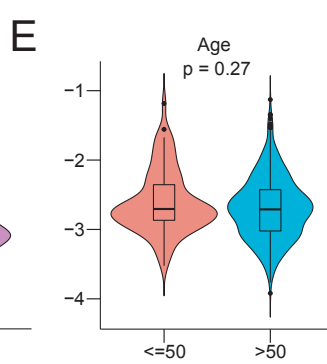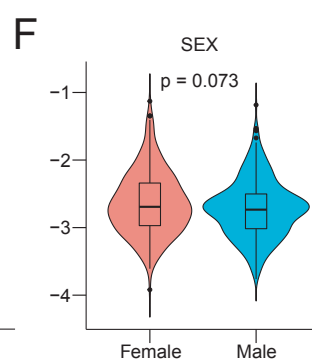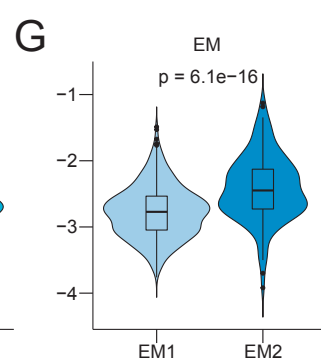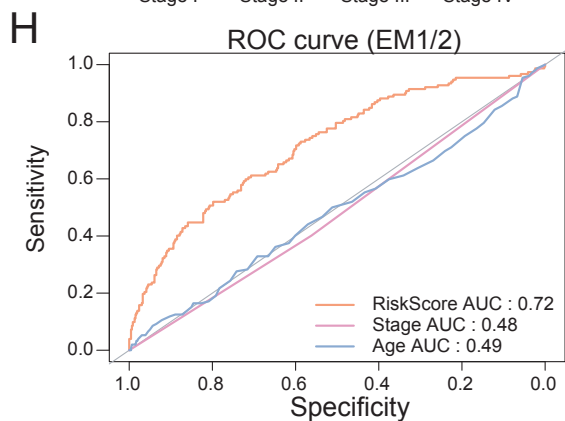

Supplement: Supplementary file 4 — Fig S4 [file JCMM-25-3194-s004.pdf]
